# A case report of intrapulmonary schwannoma

**DOI:** 10.1007/s11748-013-0233-5

**Published:** 2013-03-23

**Authors:** Takuro Yukawa, Katsuhiko Shimizu, Yuji Hirami, Riki Okita, Shinsuke Saisho, Ai Maeda, Koichiro Yasuda, Masao Nakata

**Affiliations:** Department of General Thoracic Surgery, Kawasaki Medical School, 577 Matsushima, Kurashiki, Okayama 701-0192 Japan

**Keywords:** Intrapulmonary schwannoma, Neurogenic tumor

## Abstract

A 38-year-old man without any symptoms was admitted to our institution because of an abnormal shadow found incidentally on a chest X-ray. Chest computed tomography showed a round mass in the lingular segment of the left upper lobe. Lingular segmentectomy was performed, and the histopathological diagnosis was intrapulmonary schwannoma. Immunohistochemical staining revealed a positive result for S-100 protein and negative results for CD34 and desmin. We report this case of intrapulmonary schwannoma, which is extremely rare.

## Introduction

Schwannoma may arise from any peripheral nerve and is often found in the chest wall and posterior mediastinum. However, case reports of intrapulmonary schwannoma are extremely rare. We report a case of primary intrapulmonary schwannoma.

## Case

**Fig. 1:**
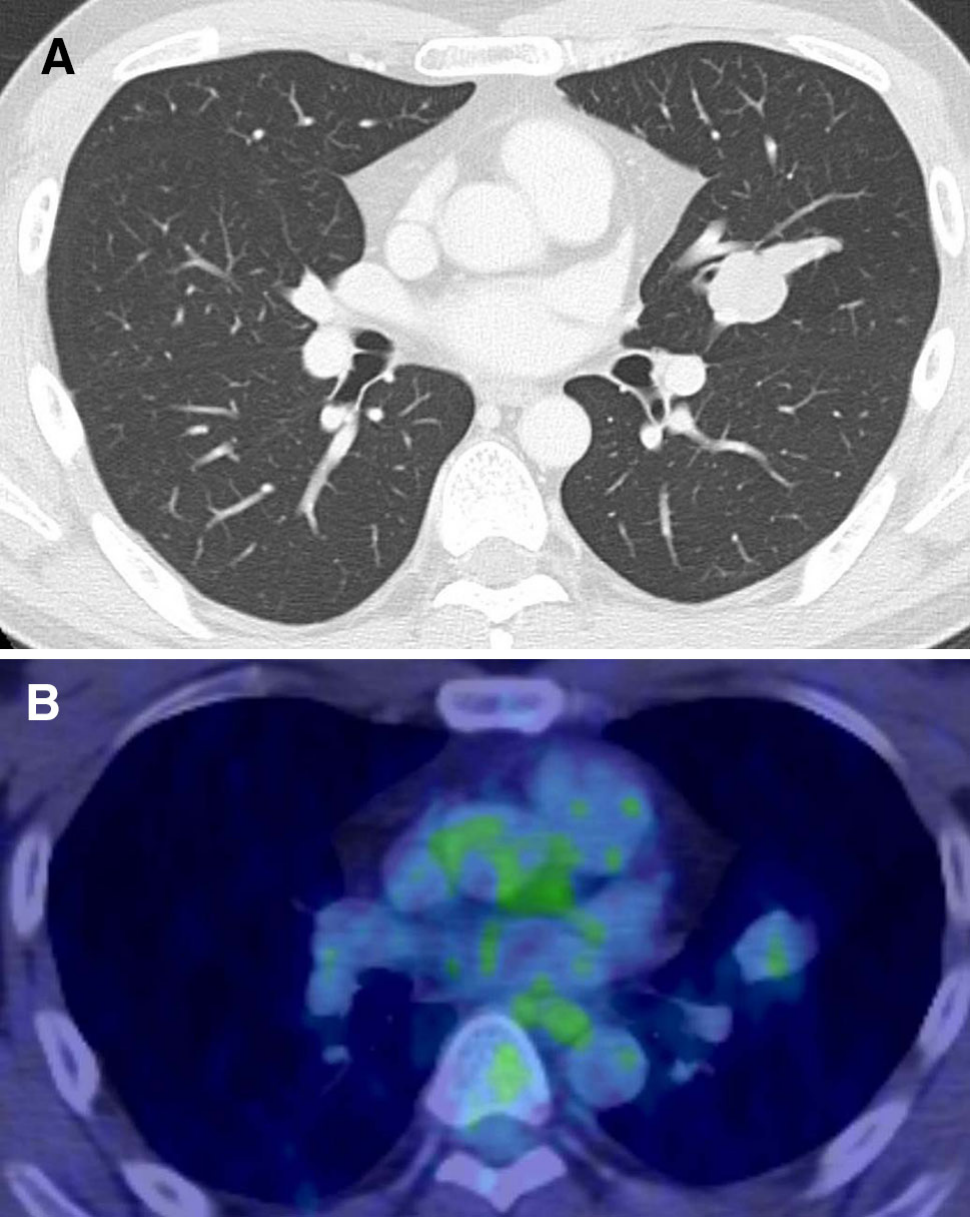
**a** Computed tomography shows a mass with well-defined margins in the lingular segment of the left upper lobe. **b** 18-Fluorodeoxy-glucose (FDG) positron-emission tomography (PET) showed no accumulation in the tumor

A 38-year-old man was referred to our institution because of an abnormal shadow found incidentally on the chest X-ray. His past medical history was insignificant. The plain chest X-ray showed a mass lesion in the left middle zone. The mass was about 3 cm in diameter. Chest computed tomography (CT) showed a round and homogeneous mass 25 × 18 mm in size with a well-defined margin in the lingular segment of the left upper lobe (Fig. 1a). 18-Fluorodeoxy-glucose (FDG) positron-emission tomography (PET) showed no accumulation in the tumor (Fig. 1b). The CT and FDG-PET findings suggested the possibility of a benign tumor or a low-grade malignancy. As a malignant tumor could not be definitively ruled out, we performed lingular segmentectomy.

**Fig. 2:**
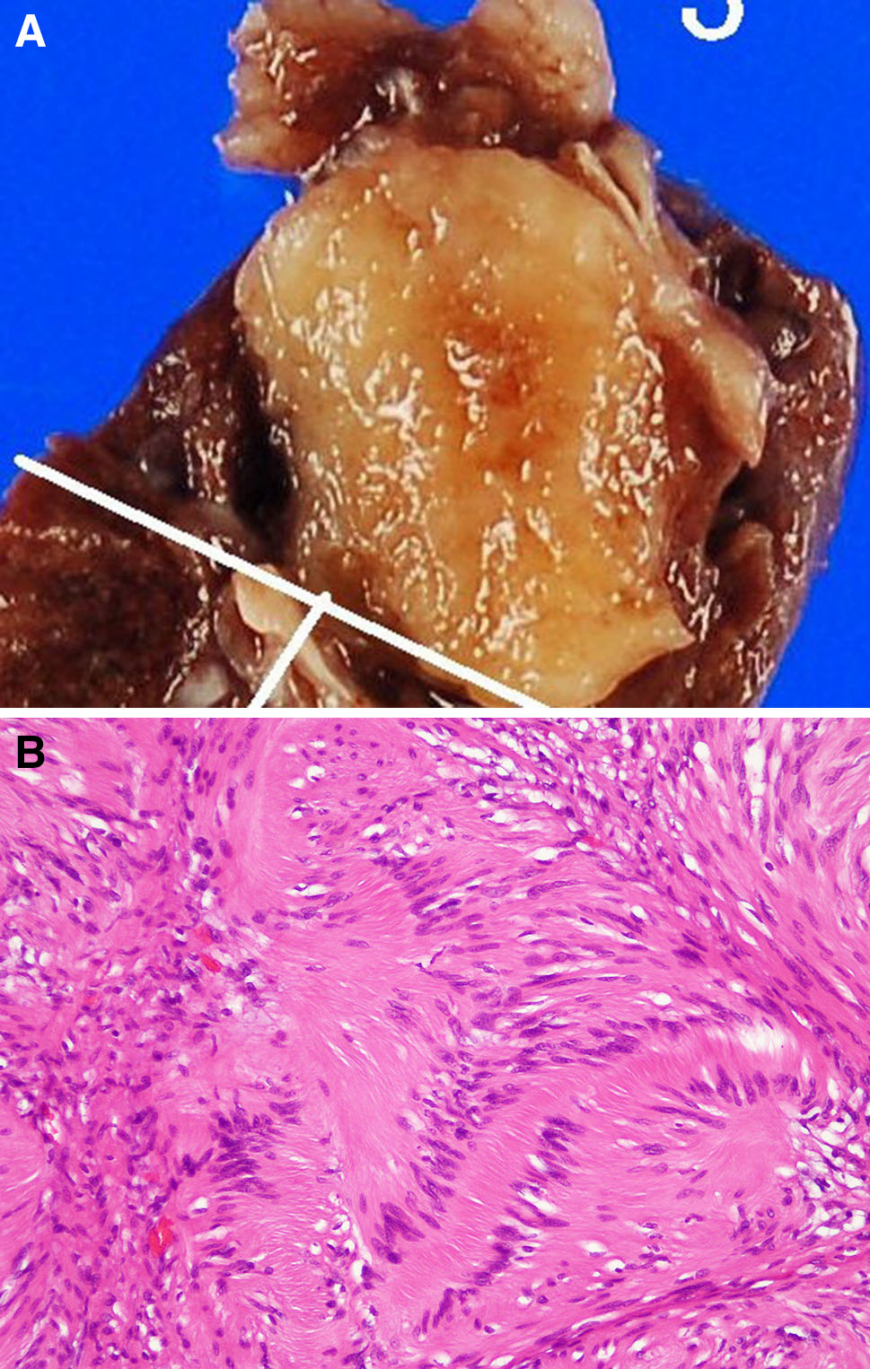
**a** Macroscopic examination of the specimen showed a round, well-demarcated tumor in the lung, without invasion of the surrounding tissues. The cut surface was yellowish-white in color. **b** Microscopic examination revealed elongated tumor cells with spindle-shaped nuclei

Macroscopic examination of the resected specimen showed a well-demarcated round tumor in the lung, without any evidence of invasion of the surrounding tissues. The cut surface was yellowish-white in color (Fig. 2a). Microscopic examination revealed proliferation of elongated tumor cells having spindle-shaped nuclei, with cellular palisading (Fig. 2b). No necrosis or nuclear atypia was observed. Immunohistochemical staining demonstrated positive staining of the tumor cells for S-100 protein and BCL2, but negative staining for CD34 and desmin. The histopathological diagnosis was intrapulmonary schwannoma.

## Discussion

Schwannoma is one of the few truly encapsulated neoplasms of the human body, and is almost always solitary. It most commonly occurs on the flexor surfaces of the extremities, neck, mediastinum, retroperitoneum, posterior spinal roots, and cerebellopontine angle [1]. However, schwannomas are extremely rare in the lung, regardless of the patient age. Ohtsuka and colleagues [2] reported 62 patients (28 men, 34 women) aged 5–83 years, with intrapulmonary or bronchial schwannomas, and stated that this neoplasm accounted for about 0.2 % of all pulmonary neoplasms. In 55 % of these patients, the tumor originated within the tissues in proximity to the terminal segmental bronchus.

On radiographic images, peripheral intrapulmonary schwannoma appears as a round mass with well-defined margins [2]. In patients with the tumor located proximal to a lobar bronchus, atelectasis or pneumonia may occur, associated with cough and dyspnea. About half of the patients have some symptoms; however, most patients with peripheral intrapulmonary schwannomas, as in the present case, have no symptoms [2].

Today, FDG-PET is a useful approach to differentiate malignant solitary pulmonary nodules from benign nodules. Beaulieu et al. reported the maximum standard uptake values (SUV_max_) of the 10 schwannomas ranged from 1.9 to 7.2, and the variation in the SUV_max_ could be explained by the variation in cellularity [3]. However, the reason why high FDG accumulation is found in benign tumors such as schwannoma remains unclear, and a diagnostic value of FDG-PET with intrapulmonary schwannoma is not established.

It is reported that in rare instances, schwannomas may undergo malignant transformation. The expression level of Ki67, a tumor cell proliferation marker, has been reported to be useful for determining the malignant potential of these tumors [4]. Kindblom and colleagues [4] compared the Ki67 expression levels in 26 malignant peripheral nerve sheath tumors and 24 benign nerve sheath tumors (schwannomas), and reported that significantly high density of nuclear staining was found in malignant peripheral nerve sheath tumors.

For the treatment of primary intrapulmonary schwannoma, surgical resection, intrabronchial resection with endoscopy, and yttrium aluminium garnet (YAG) laser resection have been employed [5–7]. Because of the low malignant potential of these tumors, tumor enucleation or partial lung resection is thought to be adequate, with lobectomy not being necessary. In the present case, we performed segmentectomy to accomplish complete resection, because we did not obtain the pathological diagnosis before the operation and the tumor was located in the central part of the superior lingular segment.

In conclusion, we had reported a case of intrapulmonary schwannoma, what is extremely rare. The symptoms and CT manifestations are nonspecific, therefore preoperative diagnosis is generally difficult.
